# Efficacy of functional foods mixture in improving hypercholesterolemia, inflammatory and endothelial dysfunction biomarkers-induced by high cholesterol diet

**DOI:** 10.1186/s12944-017-0585-4

**Published:** 2017-10-06

**Authors:** Hessah Mohammed Al-Muzafar, Kamal Adel Amin

**Affiliations:** Department of Chemistry, College of Science, University of Dammam, P.O. Box 1982, Dammam, 31441 Saudi Arabia

**Keywords:** Hypercholesterolemia, Functional food, Lipids, Cardiac, Endothelium, Inflammation

## Abstract

**Background:**

Hypercholesterolemia associated with cardiovascular diseases is a global health issue that could be alleviated by functional foods. This study aimed to explore the effects of a high-cholesterol diet on lipid profile, cardiac, inflammatory, and endothelial dysfunction biomarkers, and the possible improvement by functional foods mixture.

**Methods:**

Male albino rats weighing 100–150 g were randomly divided into four equal groups: 1st control, giving a normal diet; the 2nd received high-cholesterol diet for 8 weeks, the 3rd received the high-cholesterol diet + functional foods mixture, and the 4th administered high-cholesterol diet +atorvastatin (20 mg) orally.

**Results:**

The results showed a significant increase in lipid profile and cardiac biomarkers levels (lactate dehydrogenase, creatine kinase and homocystein), also inflammatory markers, as, tumor necrotic factor alpha and chronic reactive proteins were elevated, moreover, vascular adhesion molecule-1 and nitric oxide synthase were disturbed in high-cholesterol diet compared with normal group. While administration of atorvastatin and functional foods mixture ameliorated these alterations.

**Conclusions:**

Administration of functional foods mixture and atorvastatin were effective in treating hypercholesterolemia, reduce the risk of inflammation and cardiovascular biomarkers with a high safety margin. These efficiencies may be due to its active ingredient that improve the imbalance in the measured biomarkers.

## Background

Hypercholesterolemia is still the principal reason for illness and mortality, despite developments in primary and secondary prevention over the past few decades. Hypercholesterolemia is associated with several metabolic disorders, such as T2DM, cardiovascular diseases (CVD), and atherosclerosis, causing morbidity and death worldwide [1]. Hypercholesterolemia has a proatherogenic effect and may directly affect the myocardium, causing contraction dysfunction. Also, hypercholesterolemia affects cardiac gene expression, resulting in elevated myocardial oxidative stress, mitochondrial disturbances, and inflammation-initiated apoptosis. These disturbances may produce cardiac dysfunction and promote infarction. Both experimental and clinical research recommends that increased nitrative and/or oxidative stress has a vital effect on heart problems in hypercholesterolemic status [2, 3]. Moreover, hypercholesterolemia may be prevented and treated by mechanisms using cardioprotective compounds. Although the mechanisms of protection and treatment are not fully clarified, they involve the dysregulation of the endothelial Nitric oxide synthase (NOS) and peroxynitrite-MMP2 signaling pathways and the modulation of ATP-sensitive potassium channels and apoptotic pathways [2]. We propose other mechanisms of endothelial dysfunction, inflammatory markers and vascular adhesion molecule (VCAM-1) regulation by hypercholesterolemia and its treatments. Failure to prevent and treat hypercholesterolemia is the main threat for cardiovascular illness, adversely affecting the myocardium itself and promoting atherosclerosis [3]. Therefore, treatments and prevention strategies for harmful cardiac consequences should aim to control the myocardial oxidative stress, lipid metabolic disturbances, and inflammation associated with hypercholesterolemia.

The marketplace offers numerous kinds of hypocholesterolemic medications that may be given alone or with other treatments. Because these medications have disagreeable side effects, we need an extensive investigation into natural treatments of functional food or medicinal plant as complementary or alternative therapies [4, 5]. Therefore, there is great interest in functional foods as a potential alternative treatment for hypercholesterolemia, being particularly suitable for cases where blood cholesterol level is somewhat high (about 5.2–6.2 mmol/L) but not too high to allow the therapy of cholesterol-lowering prescriptions [6].

Common food rich in cholesterol may lead to hyperlipidemia with increased oxidative stress, which are major risk factors for various metabolic disorders. Also, the medications for treatment are not free from side effects, while the active substances in natural functional food offer an attractive alternative, due to their hypocholesterolemic, negligible side effects, acceptability and cost effectiveness. The optimal physiological metabolism and cellular functions accomplished with the help of functional foods support the body for the biochemical and physiological functions [7, 8]. The mechanism of functional foods is predicted to act through improving the availability of several vitamins, minerals, essential fatty and amino acids, probiotics and prebiotics.

Ator. is an inhibitor of 3OH,3MG-CoA reductase, widely known as statins, and usually used for treatment of hypercholesterolemia with significant efficiency. Ator is well tolerated with infrequent adverse effects, including slight gastrointestinal troubles, increased blood transaminase activities and muscle-linked illnesses. A few cases of acute pancreatitis have been reported with Ator therapy. The U.S. Food and Drug Administration have also stated pancreatitis side effects from taking Ator [9].

Statins are one of the best medication available in clinical practice for lowering hypercholesterolemia to prevent cardiovascular problems, however, many patients show muscles related side effects and statin intolerance, which might be detected in 10–15% of patients [10, 11]. Statin interruption besides non-adherence to its therapy due to cultural causes, cost, adverse side effects, and shortage of disease awareness, remain two of the highest challenges for lipidologists [12].Statin therapy can accompany with the adverse effect of muscle problems, statin intolerance as well as non-adherence and discontinuation of its therapy, frequently resulted in insufficient control of blood cholesterol levels and increased the cardiovascular risks and disease [13].Improvement of cardiovascular threat needs more active communication between patients and their physicians, apply medication (long-term adherence with high-dose statins, proprotein convertase subtilisin/kexin type 9 (PCSK9) inhibitors or mipomersen) and non-medication (Nutraceuticals, functional food, bile acid and nicotinic acid) course of the therapy [12, 13].

Fenugreek is a traditional medicinal plant with a remarkable functional nutritive outline. Fenugreek seeds have a considerable quantity of fiber, glycolipids, essential fatty acids, phospholipids, vitamins A, B1, B2 and C, niacin, and various additional functional components. It is reasonably tolerant to salinity and can be grown on peripheral lands commercially. Because of these features, it may be suitable for numerous cropping methods and cultivated successfully in various environments. Besides its medicinal usages, it may have application as an exceptional off-season food [14]. Several researchers consider it as a probable nutraceutical due to its chemical constituents. Thus, fenugreek is applied in many fields to improve artificial medications along with other more expensive therapy to treat illnesses [15]. Additional studies can be done to segregate bioactive substances from crude extracts to reflect promising avenues in the field of the advance of natural products for the treatment of diseases.

**Fig. 1:**
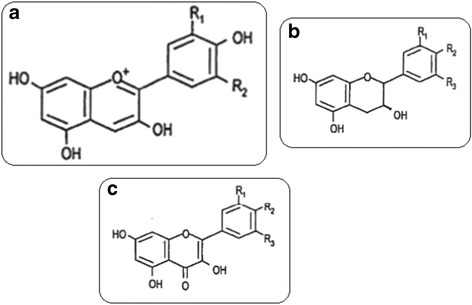
Chemical structures of flavonoids. **a** Anthocyanidins include, pelargonidi (R1, R2 = H), Cyanidin (R1 = OH, R2 = H) and Delphinidin (R1, R2 = OH). **b** Flavanol include, catechins (R1, R2 = OH and R3 = H) and Gallocatechin (R1, R2, R3 = OH). **c** Flavonols include, kaempferol (R1, R3 = H and R2 = OH), Quercetin (R1, R2 = OH and R3 = H) and myricetin (R1, R2, R3 = OH)

Concerning phytochemical structure, formerly recorded data on fenugreek indicated the presence of alkaloids, flavonoids and phenolic acids, polysaccharides, triterpenoids, steroidal sapogenins and nicotinic acid [16, 17, 18]. Identification of 32 phenolic substances in fenugreek, among which several flavonoid glycosides and phenolic acids have been recognized on the basis of their UV and MS spectra. Hydroxycinnamic acids mostly conquered by caffeic acid byproducts were also identified. The peak of the recognized composites was not acylated and acylated flavonoids with luteolin, apigenin, and kaempferol as aglycons [19]. Appropriate studies accompanied by clinical trials are needed for natural plant compounds to be applied successfully in humans [20]. Phenolic substances are present in fruits, beverages as fruit juices, berry, and vegetables [21]. Chemically, phenolics are molecules having an aromatic ring containing one or more OH groups. Polyphenols have antioxidant, metal-chelating properties, and may prevent several diseases linked to oxidative stress, cardiovascular diseases, inflammation and atherosclerosis [22, 23, 24]. Polyphenols classified depending on the carbon skeleton nature into 4 main types; phenolic acids, lignans, flavonoids and stilbenes [23]. Some of the chemical structures of flavonoids were demonstrated in Fig. 1 [25].

Tartary buckwheat (*Fagopyrum tataricum*) is a common herbal and functional food that is thought to be related to the reduced threat of Type 2 diabetes mellitus (T_2_DM). It contains large amounts of nutrients and phytochemicals such as flavonoids and D-chiro-inositol so could improve postprandial hyperglycemia and decrease fasting glucose. Protein and flavonoids from buckwheat inhibit hypercholesterolemia and recover the lipid profile to the normal [26]. Buckwheat is a cereal mostly developed in cold and hilly areas. Buckwheat seeds have great nutritional value and protective properties against several types of diseases. Buckwheat has antimicrobial, trypsin-inhibiting, anticancer, anti-hypocholesterolemia and anti-diabetic properties because of its proteins and enzyme hydrolysates. It was able to inhibit lipid oxidation in mouse brain lipids and the antioxidant capacity and activity of buckwheat may be increased by an increase in availability of antioxidant constituents, such as rutin and quercetin [27]. Buckwheat (BW) considers as a pseudocereal free from gluten and a member of Polygonaceae family. BW grain offers a wide-ranging of useful effects and provides a highly nutritional food constituent.

The endothelium has several functions including, vascular tone and permeability regulation, fibrinolysis and coagulation, immunological and inflammatory reactions and cell growth. Modifications of any of these activities may induce a vascular endothelial malfunction. Nowadays dysfunction of the endothelium is extensively accepted as an initial biomarker of cardiovascular disturbances, making it, comprehensive prevention or therapy, a developing, motivating therapeutic goal [28]. One of the Therapeutics target to maintain endothelial function is pravastatin or Ator treatments that lower cholesterol level via different mechanisms improving endothelial function [29]. Also, statins play a significant role in treating endothelial dysfunction by reducing the adhesion of inflammatory cells, where inflammatory pathways have a vital role in the growth and development of atherosclerosis [30]. Alternative therapy beside Ator resulted in enhancement in endothelial function and a lessening in inflammatory biomarkers in patients with steady cardiovascular disease [31].

Update research found that berries can play a valuable influence on decreasing low-density lipoproteins (LDL) and its oxidation. Insufficient data mentioned that berries could have a promising action on endothelial healthiness and blood pressure. Collectively, it is recommended to integrate products prepared with functional foods as berries into a cardiac-healthy nutrition [32].

Supplementary safe, cheap and accessible approach to hypercholesterolemia treatment may be functional foods that contain bioactive compounds having some health-promoting action and possess antioxidative or anti-inflammatory and hypolipidemic activities [33, 34, 35]. Phytochemicals comprise phenolic substances, as flavonoids e.g. quercetin and epicatechin [36]. Some species of this food include, fenugreek extract, buckwheat, has antioxidant, hypocholesterolemic properties. The functional foods mixture (FFM) thus may reveal the favorable therapeutic potential for the treatment of hypocholesterolemia and act as a pharmaceutical agent. Combination therapy using FFM provides several active principles for cardiovascular disease treatment, in addition it is important to mention that in fact combination therapy is a clear answer to the lack of medication adherence and poor compliance, as well as to statins intolerance. Adherence assumes the patient’s agreement with the approvals, while compliance suggests patient inactivity. Both expressions are challenging in describing medication -taking behavior as they overstress the doctor’s control through the course of providing medications [37]. Overcome of medication-taking behavior issues through combination therapy will avoid assigning responsibility completely to the patient and support in recognizing active therapy and complete benefits of the treatments [38].

The beneficial role of these functional foods combination provides us the tool to think and design our study and use some of them related to cardiovascular disorders. So, one of our aims in this project was to study the effects of some functional food in a mixture, on lipid profiles, cardiac and proinflammatory biomarkers in rats fed high-cholesterol diet (HCD). Therefore the objective of the current work was to, induce a model of hypercholesterolemia and explore the role of some functional foods mixture (Fenugreek, Buckwheat, and Berry) in maintaining a healthy metabolism and explore the role of hypercholesterolemia in lipid, cardiac, endothelial dysfunction, inflammatory biomarkers and possible improvement by these FFM. Moreover, demonstrate the potential roles and mechanisms of the tested FFM comparing with Ator in the treatment of hypercholesterolemia. Furthermore, the current experimental study aims to have the results potentially reassigned to human studies and a possible clinical application will be planned for forthcoming studies on human to be continued.

## Methods

### Materials

#### Diet

Two types of diets, control rat chow diet and high in cholesterol (HCD).

The control diet included a homogeneous mixture of 100% rodent diet; the high-cholesterol diet (HCD) composed of control diet with 2% cholesterol and 0.4% bile acid. This diet was used for induction of hypercholesterolemia in rats. Control diet was composed of 65% carbohydrates, 5% fat, 20% crude protein, 5% minerals and vitamins, and 5% dietary fiber. The HCD consisted of 60% carbohydrates, 7.6% fat, 2% cholesterol, 0.4bile acid, 20% crude protein, 5% minerals and vitamins, and 5% dietary fiber.

#### Experimental animals

One hundred rats weighted 100–150 g were supplied by institute for research and medical consultation (IRMC). Rats were kept under observation for one week before the onset of an experiment to be acclimatized and housed individually in plastic cages at room temperature 24 ± 3 °C, less than 12 h light/dark cycle in the laboratory of IRMC, University of Imam Abdulrahman Bin Faisal (University of Dammam).

#### Medications for treatment

Atorvastatin: It used by dissolving 20 mg in 150 ml DW, each rat giving 1 ml per Os in a dose of 0.666 mg/kg (0.1332 mg/rat/day by stomach tube). Ator purchased from Jamjoom Pharmaceuticals Co. Ltd., Jeddah, Saudi Arabia. Ator used as reference medication for cholesterol therapy to compare with FFM.

Functional food mixture: The mixture composed of Fenugreek was available as a powder from GNC Holdings, Inc., Pittsburgh, PA and giving in a dose of 61 mg/kg, Buckwheat available as a dry bean purchased from local markets in Dammam City and giving in a dose of 25 mg/kg and Cranberry was available as powder purchased from GNC Holdings, Inc., at a dose of 53.33 mg/kg. These functional food mixed in a ratio of 1:1:1 then the mixture administrated as suspension per Os using a stomach tube.

### Methods

#### Preparation of FFM

The extraction of buckwheat was done by the following method: 50 g of the ground seeds was subjected to a 625-ml hot water (70-80c) extraction for 4 h with mixing. The suspended solution was filtered using two layers of cheesecloth and a filter paper. The extract was centrifuged for 10 min at 2000 g. on 4 °C. Then concentrated using Freezdryer to get buckwheat powder. The fenugreek and Cranberry used as powder. 0.61 g of the Fenugreek and 0.533 g of CranBerry were weighted with 0.25 g of Buckwheat powder and 50 ml distilled water. These weights were calculated based on the determined doses for each of the used nutrients, buckwheat (25 g/kg), fenugreek (61 mg/kg), cranberry (53.33 mg/kg). The FFM kept in the refrigerator and daily given to the rats during the experiment (4 weeks) which is the treatment period.

#### Experimental design and animal grouping

Our experiments proceed for 12 weeks and include induction of hypercholesterolemia for 8 weeks and treatment period extends for 4 weeks. A total of 100 rats were used for this study and classified into five equal groups as follows: 1st control group receiving standard diet; 2nd high-cholesterol diet (HCD) diet for 8 weeks for induction of hypercholesterolemia; 3rd HCD with functional food mixture and 4th giving HCD with (20 mg) Ator.

#### Blood sampling

Blood were collected from medial, canthus of the eye, via microhematocrit capillary tube at fasting state. The blood samples were collected in dry glass centrifuge tubes, allowed to coagulate at room temperature and centrifuged at 3500 rpm for 20 min for separation of serum. The non-hemolyzed supernatant sera were separated using clean, dry disposable plastic syringes and stored at −80 °C for subsequent biochemical measurements.

#### Biochemical analyses of serum

Blood samples used to carry out the following biochemical analysis: lipid profile include triglyceride (TG), Cholesterol, Low-density lipoproteins (LDL), high-density lipoproteins (HDL), lactate dehydrogenase activity (LDH), creatine kinase (CK) and homocystein were determined by kits provided from TECO diagnostic, 1268 N. Lakeview Ave. Anaheim, CA 92807. Also, inflammatory markers, C-reactive protein (CRP), Tumor necrotic factor (TNFα) and Nitric oxide synthase were analyzed using ELISA kits, catalog Number:MBS281245, MBS355371, and MBS702741 respectively, and provided from MyBioSource, Inc. VCAM-1 Quantitatively determined using ELISA kits in serum provided from MyBioSource, Inc. P.O. Boxes 153,308, San Diego, CA 92195–3308, USA (Catalogue No MBS703064). VCAM -1 assay was as follows; Use the measurable sandwich EIA. Antibody precise for VCAM-1 coated the microplate. Serum and standard are pipetted into the wells and the halted antibody bound the VCAM-1. Once removing any boundless ingredients, antibody conjugated to biotin specific for VCAM-1 is inserted into the wells. Washing then added to the wells Horseradish Peroxidase conjugated with avidin. After washing to eliminate boundless avidin-enzyme substance, The substrate is inserted into the wells resulting in developments of color in proportional to the amount of VCAM-1 in the 1st stage. Stop the color and its strength is determined at 450 nm using a microplate reader. The detection rate was 15:1000 ng/ml. This analysis has great sensitivity and outstanding specificity for identification of rat VCAM-1.

Estimation of FFM safety and toxicity: A program was used to evaluate the safety of FFM. FFM was given orally by gavage at 5 and 10 times of the applied original dose daily for a week to a group of 8 male rats and another 8 rat giving saline act as a control to examine its safety and toxicity. After administration, the time of survival for each rat was recorded for 24 h and the animals were noticed to report the health changes and mortality for each [39].

### Statistical analysis

Data were analyzed using one-way analysis of variance (ANOVA) followed by Tukey-Kramer methods for *post-hoc* analysis. A value of *p* < 0.05 was considered statistically significant. Graph Pad Prism 6 software (San Diego, CA, USA) was used for statistical analysis. Data were presented as mean ± SEM.

## Results

During the experiment, no pathological changes were observed in the appearance or behavior of the animals. Mean body weight gain, increased in rats on the HCD compared with controls (Table 1).

**Table 1:** Effect of FFM and Atorvastatine on body weight measurement HCD fed rats

	Normal	HCD	HCD + FFM	HCD + Ator
Initial Bwt	137.83 ± 4.52	141.25 ± 2.22	132.17 ± 2.1	136 ± 3.22
Mean Bwt gain	7.82 ± 2	9.49 ± 2.08*	6.16 ± 3.08	4.34 ± 2.99

These values represent means and standard errors, * indicate significant variations at *P* > 0.05

**Fig. 2:**
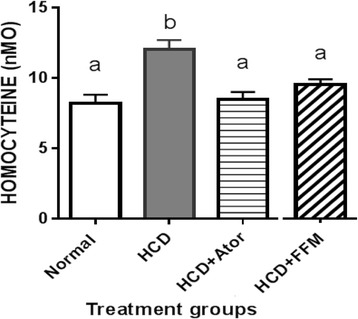
Effect of FFM and Atorvastatin extract on homocysteine (nMo) level in HCD fed rats

Table 2 showed that HCD induced significant hypercholesterolemia and elevation in LDL, LDL/HDL ratio, CK-MB, and homocystein (Fig. 2) serum levels, However, there were slight changes in TG, HDL and LDH serum levels in comparison with normal groups. Supplementary administration of FFM or Ator, ameliorate these deviations with different grades (Table 2) in the same significances with minor differences in their concentration, indicating the effectiveness of FFM compared with Ator. Our data exhibited significant variations in their serum lipid profiles. The HCD-fed rats showed a significant increase in the levels of serum TC and LDL (*p* < 0.01), and a decrease in the LDL/HDL ratio compared with the normal diet-fed rats (Table 2). Supplementary administration of Ator, and FFM significantly improved these deviations with different grades (Table 2).

**Table 2:** Effect of FFM and Ator. on lipid profiles and cardiac biomarkers in HCD fed rats

	Normal	HCD	HCD+ FFM	HCD + Ator
TC (mg/dl)	61 ± 2.5 a	87 ± 4.3b	72 ± 3.1c	73 ± 3.0c
TG (mmol)	1.2 ± 0.07	1.3 ± 0.05	1.6 ± 0.18	1.2 ± 0.05
LDL mg/dl	2.2 ± 0.19 a	3.2 ± 0.18b	2.5 ± 0.23 a	2.4 ± 0.14a
HDL(mg/dl)	61 ± 2.8	64 ± 2.1	64 ± 1.4	60 ± 2.9
LDL/HDL ratio	3.6 a	5 b	3.9 a	4 a
LDH (ng/ml)	5.1 ± 0.35	5 ± 0.26	4.7 ± 0.25	5.1 ± 0.4
Homocystein (nMO)	8.2 ± 0.60	12 ± 0.67*a	9.5 ± 0.40 b	8.5 ± 0.53 b
CK-MB (ng/ml)	0.47 ± 0.13	0.72 ± 0.096	0.8 ± 0.12	0.56 ± 0.15

These values represent means and standard errors, the different superscript letters mean a significant difference at P > 0.05

Table 3 demonstrated significant rises in TNFα and CRP as inflammatory markers, while there were significant increases in Nitric oxide synthase (NOS) and VCAM-1 as an endothelial function markers in HCD compared with normal group. However, giving FFM or Ator balance and normalized these alterations.

**Table 3:** Effect of atorvastatin, and FFM on inflammatory and endothelial function biomarkers in HCD fed rats

	Normal	HCD	HCD + FFM	HCD + Ator
CRP (ng/ml)	0.2 ± 0.02^a^	0.46 ± 0.14^b^	0.25 ± 0.054^a^	0.35 ± 0.054^b^
TNFα (pg/ml)	13 ± 6 ^a^	20 ± 11 ^b^	2.8 ± 0.92^c^	4.8 ± 2.5^c^
NOS (μIU/ml)	168 ± 9.2^a^	214 ± 8.6^b^	192 ± 8.9^a^	189 ± 7^a^
VICAM1 (pg/ml)	61 ± 4.5^a^	70 ± 3.6^a^	30 ± 9^b^	72 ± 4.7^a^

These values represent means and standard errors, the different superscript letters a, b, c, indicated a significant difference at P > 0.05

**Fig. 3:**
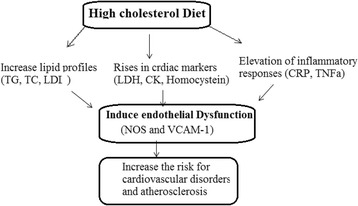
Proposed mechanisms and biochemical reactions in response to HCD in rat

Figure 3 showed planned mechanisms and biochemical reactions in response to HCD in rat that indicated rises in lipid profile, cardiac and inflammatory biomarkers that resulted in endothelial dysfunction and consequently, increase the progress of cardiovascular diseases.

Evaluation of the FFM safety program was not linked with mortality and seemed to be well tolerated up to 10 times. There were no toxicological observations, no dead rats and did not show any symptoms of diarrhea, nervous and behavioral, feed and water consumption or significant changes in body weight gain in rat group. There was no adverse side effect with the highest 10-time dose. These results indicated the possible usage of FFM as a component of functional food.

## Discussion

### Role of FFM and Ator on hyperlipidemia and cardiac function biomarkers

The current study showed significant changes in the mean body weight gain in HCD-fed rats in comparison with control groups. This result was in agreement with other studies [40]**.** Rat consuming the cholesterol diet showed more weight gain than the control group. In the present study, HCD induced higher body gain that, accompanied by disturbances in lipid profiles as explained by higher triglycerides, total cholesterol and LDL and a decrease in the level of serum HDL (Table 2). Administration of our formula of FFM ameliorates these changes in comparison with HCD group. The action of FFM may be due to its contents of active compounds existing in FFM that contain buckwheat (BW), fenugreek and berry. Buckwheat as one of the components of the FFM contains a lot of soluble fibers that can decrease the risk of cardiovascular illness. The lowering effect of buckwheat for lipids and cholesterol levels is because of its beta-glucan as an active component of soluble fiber [26, 35, 41] and the effect of sapogenins as hypocholesterolemic in fenugreek [18, 42].

Several studies have established that nutraceuticals and function food have possible lipid-lowering properties, safety and high tolerance, however, strong, durable clinical trials to study the roles of nutraceuticals on clinical outcomes are still required [43, 44], it has to be obviously stated that still no results evidencing that nutraceuticals can avoid CVD mortality or morbidity [35].

Many countries consumed bread based mainly on wheat and separate the bran from the wheat, therefore, the bread lacks fiber, vitamins, and minerals which are important for a healthy heart. The BW extract supports health due to its antidiabetic, anti-inflammatory, neuroprotection and antitumor properties. Furthermore, BW has been described to have antioxidant and prebiotic activities. The animal experiments and *invitro* recommend that bioactive substances of BW like, D-chiro-inositol, flavonoids (mostly rutin and quercetin) and proteins may be accountable for the previous roles [45].

Various natural peptides separated from BW seeds were evaluated to have many functional and useful effects, as antibacterial, anticancer, hypotensive and antioxidant properties. In addition to trypsin preventing activity on proteases, BW trypsin inhibitors similarly showed antibacterial action on fungi, gram +ve and -ve bacteria and anticancer action for many types of tumor cells. These actions are linked to the specific peptide molecules active site, whereas the hypolipidemic effects and the hypotensive action are most possibly correlating with buckwheat proteins that contain distinctive amino acids, and minor peptides retain the appropriate action. The peptides of buckwheat display potential use in the area of functional food and traditional medicine investigation [46].

In agreement with our results of FFM, Sharma and Choudhary [47] reported that the aqueous emulsified fenugreek seeds powder reduced significantly the TC, LDL-C, and the atherogenic index, with rise in the HDL-C in experimentally hyperlipidemic rabbits as compared to control group, whereas there were non-significant differences in the triglyceride, VLDL-C, and body weight of both groups. It was concluded that fenugreek seeds powder had a significant hypolipidemic effect in the experimentally-induced hyperlipidemic group. Fenugreek as a functional food has an anti-peroxidative effect which may be due to its regulatory effect on plasmatic lipid metabolism [33]. In addition, both fenugreek and Ator have hypolipidemic action in rabbits, however Ator is more potent than fenugreek powder [34].

Cardiovascular indices of TC, LDL, LDH and homocystein activities (Table 2 and Fig. 2) were elevated in HCD compared with normal groups. These data indicated an association of HCD with cardiovascular markers disorders. FFM ameliorated the effect of HCD due to its active ingredient.

Growing evidence suggests that intake of berry fruit has an essential role in the avoidance and treatment of greatest risk factors associated with metabolic syndrome and its cardiovascular problems in human. These actions may be due to the incidence of polyphenols, as anthocyanidins, proanthocyanidins, flavan-3-ols and phenolic acids that have antioxidant and anti-inflammatory effects [48]. The principal phenolic acids of berries are hydroxybenzoic and hydroxycinnamic acid derivate [49].

Current suggestion specifies possibility of a diet rich in berry in regulating the danger of prolonged diseases. Significant ingestion of berries improves post-prandial glycemic reaction, progresses of blood inflammatory biomarkers and rises the antioxidant ability of the blood. Long-time consumption of berries and its products may improve serum lipid profile, diminish prolonged inflammation and maintenance cardiovascular well-being, especially in people with baseline metabolic profile of increased risk for metabolic syndrome [50]. Further studies should be focused on investigating the probability of berries and berry byproducts in opposing the stress, helping healthy aging and enhancing gut healthiness.

### Role of FFM and Ator on inflammatory and endothelial dysfunction biomarkers

The inflammatory markers include CRP and TNFα. CRP is a protein of an annular pentameric type, initiated in serum and produced by hepatocytes in response to factors released by adipocytes and macrophages. Its levels increase in case of inflammation and it is considered as a protein of hepatic origin in acute-phase. Its biological function is to bind to lysophosphatidylcholines present on the surface of dead cells to stimulate the complement system.

Both TNFα and CRP were increased in HCD group, indicating the role and sequences of these mediators in the events of lipid metabolic disturbances. The current data showed that homocysteine and VCAM-1 were elevated significantly in HCD compared to the normal group. Surprisingly, NOS activity was elevated in HCD compared to that in the normal group. These data indicated very early the endothelial dysfunction and physiological mechanism of protection by increasing the activity of NOS as a vasodilator, during the period of giving HCD for protection against hypercholesterolemia.

Several illnesses such as hypercholesterolemia, hyperglycemia, inflammation, homocysteinemia, hypertension, smoking and aging occur due to the progress of endothelial malfunction. The mechanisms of the endothelial dysfunction involve increased ROS, RNS, stimulation of immune and inflammatory reactions, decreased endothelial vasodilators, increased endothelial vasoconstrictors, and imbalance of coagulation and fibrinolysis. Especially for cardiovascular diseases, endothelial malfunction involves deterioration in nitric oxide (NO) bioavailability as it plays varied biological roles, including vasodilation, anti-inflammatory, antiproliferation and antiplatelet aggregation [51].

The VCAM-1 is a protein programmed by the VCAM-1 gene in humans [52]. The VCAM-1 gene has 6 or 7 immunoglobulin domains, and is present on both small and large blood vessels, especially when the endothelial cells are motivated by cytokines (TNF, IL6, and CRP). VCAM-1 acts as a cell adhesion molecule. The VCAM-1 protein facilitates the adhesion of leukocytes to the endothelium of the blood vessels and functions in endothelial cell signal transduction for leukocytes. Also, it may have a role in the progression of rheumatoid arthritis and atherosclerosis. In reply to increased inflammatory biomarkers, CRP, TNF-α and IL6, endothelial cells VCAM-1 were upregulated that occur in HCD group.

The VCAM-1 induce monocyte adhesion. This adhesion was also induced by proinflammatory cytokines, such as 1 L-1β and TNF-α. These cytokines were induced by CRP protein produced as a result of the IL-6 response by protease activated receptor signaling, uptake of ox-LDL in arteri intima [53]. IL-6 secreted from activated-macrophage has a vital role in inflammatory response and leads to phebric and known as pyrogen endogen [54]. Moreover, cytokines may disturb the immune system and stimulate inflammation that induces damaging of the tissue [55].

The important event in the development of atherosclerosis depends on the differentiation of monocytes to macrophages. An understanding and identification of this mechanism of differentiation and its regulation may aid in the identification of novel treatment schemes. Suppression of this concept will make the leading line of protection in the avoidance and therapy of atherosclerosis [56]. The endothelium protects and regulates the vascular homeostasis via, vasodilation, inhibition of smooth muscle cell growth, and suppression of inflammatory reactions.These effects are facilitated by nitric oxide, as endogenous vasodilator and inhibitor for LDL oxidation. A fault in the action or production of nitric oxide may result in endothelial malfunction.

Significant elevation in serum levels of LDL, VCAM1 and homocysteine, alongside with rise in CRP and TNFα levels, could be identified in HCD compared with the control group. These alterations increased the potential risk of endothelial dysfunction and cardiovascular problems. However, NOS activity was opposing that dysfunction, The activity may act as a way for protection. We suggested that the imbalance and the ratio of these biomarkers are the cause of endothelial dysfunction and subsequent cardiovascular disorders. This evidence indicated that endothelial malfunction is a primary biomarker for atherosclerosis, and could be identified before alterations in the structure of vascular wall seeming on ultrasound examination or angiography. These risk aspects causing endothelial malfunction may predispose to cardiovascular disease and atherosclerosis.

The current study indicated that Ator, an inhibitor of HMG-CoA reductase, can ameliorate endothelial malfunction in HCD group, which could be due to their effect on lipids profiles, VCAM1, NOS, and homocystein. Investigations have revealed a possible mechanism for statin therapy through improving endothelial malfunction, comprising stimulation of NO activity, or production and maintenance of antioxidant properties [57]. Therefore, Ator and FFM provide anti-inflammatory, normalizing endothelium disturbances for the hypercholesterolemic group and prevent the risk factors for atherosclerosis. Serum homocysteine and VCAM1 are considered risk factors for vascular disease and atherosclerosis. An interesting aspect of the present study was that the VCAM1 increased in HCD, and FFM induced lowering effect compared with the normal and the other treatment groups (Table 3).

The HCD produces an elevation in lipid profiles and cardiac index, resulting in stimulation and disturbance in NOS and IL6 as inflammatory markers that, accompanied with VCAM accumulation in the endothelium of blood vessels of the cardiovascular system and increased the incidence of arteriosclerosis. HCD induced hypercholesterolemia and hypertriglyceridemia that improved by Ator and FFM. Ator repressed hypercholesterolemia by inhibiting HOMGCoa reductase. The current data revealed that feeding with the HCD resulted in elevations in both cardiac and vascular index of LDL, LDL/HDL ratio, homocysteine, and VCAM1. The current data provided evidence about the improvement of cardiovascular function, lipid profile in HCD feeding groups by administration of Ator or FFM, and these effects dependent on activation of NOS and inhibition of VCAM-1, Homocystein.

## Conclusions

It could be concluded that feeding HCD could initiate cardiovascular disorders via increasing lipid profile and disturbing the metabolic markers (LDH, NOS, VCAM1) that associated with disturbances in inflammatory biomarkers. This mechanism is a role for suitable operating drugs for competing cardiovascular problems. In this concern, administration of FFM ameliorated the elevated cholesterol, LDL, homocysteine, and VCAM1 in HCD-induced hypercholesterolemia, this effect may be due to the active ingredient of FFM via correcting the imbalance in the measured markers. These effects equivalent with that of Ato. as reference medication. The FFM has an anti-inflammatory role in a model of HCD through reducing TNF**α** and CRP levels. Altered Lipid metabolism associated with inflammatory biomarkers pathways and imbalance in vascular indices provides a concept on the changes due to HCD treatment and its mechanism and subsequent evaluation of treatments. The beneficial effects of FFM and Ator on inflammatory markers and dyslipidemia, may act as a natural anti-inflammatory mediator and reduce morbidity and mortality in cardiovascular problems and has a wide range of safety.
